# Global trends in hemophilic arthropathy research: a bibliometric and visualization analysis

**DOI:** 10.3389/fmed.2025.1556906

**Published:** 2025-04-16

**Authors:** Zhihao Wei, Lijun Ou, Sheng Chai, Dongdong Zhang, Gangjian Tang

**Affiliations:** Guilin Municipal Hospital of Traditional Chinese Medicine, Guilin, Guangxi, China

**Keywords:** hemophilic arthropathy, bibliometrics, visualization analysis, VOSviewer, CiteSpaces

## Abstract

**Background:**

Hemophilic arthropathy (HA), a common complication of hemophilia caused by recurrent hemarthrosis, significantly impacts patients’ quality of life. Despite ongoing research, a comprehensive overview of research trends for HA is lacking. Therefore, this study utilizes bibliometrics and knowledge mapping techniques to visually analyze the current status and developmental trends of HA-related research, and analyzed and predicted future research hotspots.

**Methods:**

A bibliometric analysis was conducted using the Web of Science Core Collection database. Data on publications, author names, countries, research institutions, journals, and keywords were extracted and visualized using Bibliometrix, VOSviewer, and CiteSpaces.

**Results:**

The number of HA-related publications has increased steadily over time. United States was found to be the leading country in terms of publications and international collaborations. HEMOPHILIA was found to be the most influential journal and Hospital Universitario La Paz to be the leading institution conducting HA-related research. Rodriguez-Merchan EC was identified as the most prominent researcher in the field. Keyword analysis identified five main research clusters, namely, quality of life and management, pathogenesis, classification and functional assessment, replacement surgery, and ankle arthritis treatment.

**Conclusion:**

This bibliometric analysis provides a comprehensive overview of the research trends for HA. Future studies must focus on elucidating the underlying mechanisms of HA, developing early diagnostic biomarkers, and exploring personalized treatment strategies to improve patient outcomes. Our study offers valuable insights to researchers to facilitate the identification of emerging trends and prioritization of future research directions.

## 1 Introduction

Hemophilia is an X-linked, inherited bleeding disorder attributable to the complete or partial lack of clotting factor VIII (hemophilia A) or factor IX (hemophilia B) (1) in which patients experience recurrent bleeding due to the deficiency of clotting factors (2, 3). Current studies indicate that approximately 80% of nontraumatic (spontaneous) bleeding in hemophilia occurs within joints (4). Recurrent intra-articular hemorrhage can cause chondrocyte apoptosis, localized synovitis, and synovial hypertrophy, resulting in progressive damage to several joints such as the elbow, knee, and hip, subsequently leading to the development of hemophilic arthropathy (HA) (5), especially of the knee joint, which is the most common and accounting for approximately 63.4% of cases (6, 7). The main clinical manifestations of HA include joint dysfunction, deformity, stiffness, and chronic pain (8), all of which can ultimately lead to the loss of joint function, osteoporosis, muscle atrophy, and permanent disability, thereby severely decreasing patients’ self-care ability and quality of life (9).

As medical technology continues to advance in recent years, HA has been increasingly studied. Findings related to the pathophysiology, pathogenesis, and diagnosis of HA have been updated based on imaging and serologic indices, potential therapeutic targets, and disease management (10, 11). However, to the best of our knowledge, no study has systematically researched this field using bibliometrics analysis. This gap in the literature indicates a research area that warrants comprehensive analysis and review.

Bibliometric analysis uses mathematical and statistical methods to qualitatively and quantitatively review and analyze research in a specific field over a specific time period (12) to systematically sort and analyze a large amount of data and reveal cutting-edge dynamics, development trends, and research hotspots in the field (13, 14). This study utilized bibliometrics and visualization analysis to provide a comprehensive and systematic review of the current research status in the field of HA globally from January 1, 1975, to October 31, 2024, bridging the gap in the field of bibliometric analysis. Our findings also shed light on the current research status, research hotspots, and ongoing trends in the field to provide a reference for HA research and clinical practice.

## 2 Methods

### 2.1 Data sources and search strategies

The Web of Science Core Collection (WOSCC) is a comprehensive, standardized database that is widely used in the academic world (15). It contains more than 12,000 high-quality journals and comprehensive citation records that provide comprehensive information, including authors, affiliations, and citations (16). Therefore, WOSCC was chosen as the target database. The search period was set from January 1, 1975, to October 31, 2024, and the relevant literature was exported and subsequently analyzed by software tools. The search period was set from January 01, 1975, to October 31, 2024, and the relevant literature from the search was exported to WOSCC. Search strings: (topic search [TS] = [hemophilic arthropathy] OR TS = [hemophilic arthropathy]; OR TS = [hemophilic arthropathies] OR TS = [hemophilic arthropathies]; OR TS = [hemophilic arthritis] OR TS = [hemophilic arthritis]; OR TS = [hemophilic osteoarthropathy]). Publication types were limited to articles and reviews, excluding retractions, withdrawn publications, and book chapters. The language was limited to English. Relevant publications were abstracted and saved. To minimize bias, all searches for the bibliometric analysis were conducted on the same day.

### 2.2 Software tools and data analysis

Bibliometrix R package (17) (R4.4.1) is a commonly used bibliometric analysis tool for quantitative analysis. Bibliometrix was used in this study for visual analysis of the journal’s H-index, author distribution, H-index and production over time, and co-citation analysis of the references.

VOSviewer is mainly used for bibliometric network analysis (18). VOSviewer 1.6.20 was primarily utilized in this study for the visual analysis of co-authorship analysis of countries, co-authorship analysis of authors, co-authorship analysis of organizations, co-citation analysis of journals, and co-occurrence analysis of keywords. VOSviewer software-specific parameters: ① Counting method: Full counting; ② Network types and node definitions: Co-authorship networks: Authors, Organization and Country; Co-occurrence networks: author keywords; Co-citation networks: cited sources. Unspecified parameters were maintained at their default configurations.

CiteSpace is the most widely used bibliometric analysis software (19). CiteSpace 6.3.R1 Pro was used in this study for the visual analysis of country distribution and collaboration, co-citation analysis of authors, citation burst of cited references, and citation burst of keywords. CiteSpace software specific parameters: ① Time Span: 1975–2024 (time slicing = 1 year); ② Node types: Country, Keyword, Cited Authors, Cited References; ③ Selection Criteria: Top *N* = 50 per slice. ④ Pruning setting parameter is set to Pathfinder and Pruning sliced network. Unspecified parameters were maintained at their default configurations.

Microsoft Excel 365 was used in this study to indicate publication and citation trends in the literature over the years. Furthermore, the online bibliometrics website^1^ was used to visualize international collaborations. This study used EndNote X9 software to count the total number of articles on different joint types involved in HA onset (detailed analytical workflow is provided in the Supplementary material). All raw data used in this study were obtained from public databases; therefore, ethical review was not required.

## 3 Results

### 3.1 Analysis of the annual volume of publications

**Figure 1:**
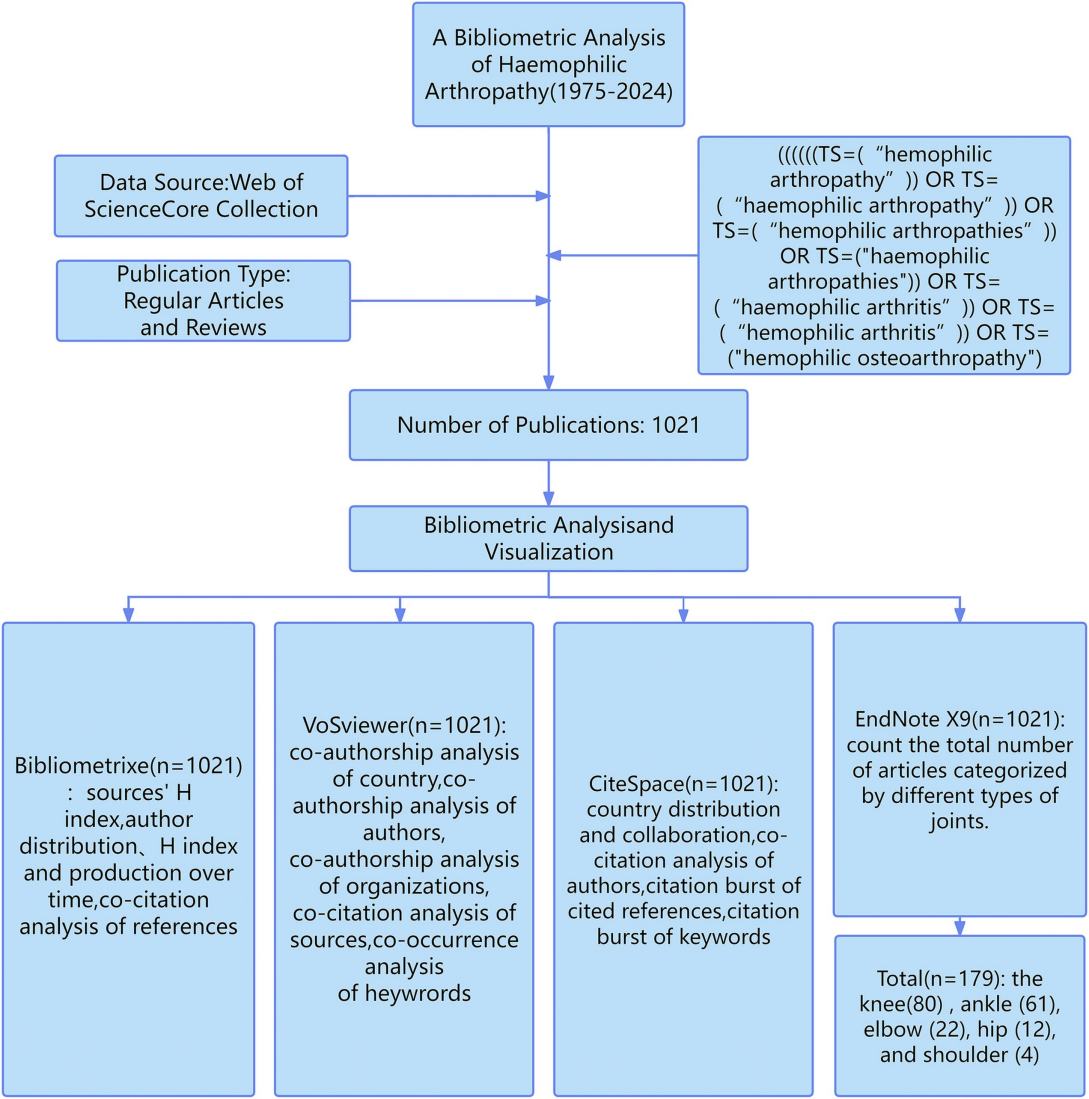
Workflow of the study.

**Figure 2:**
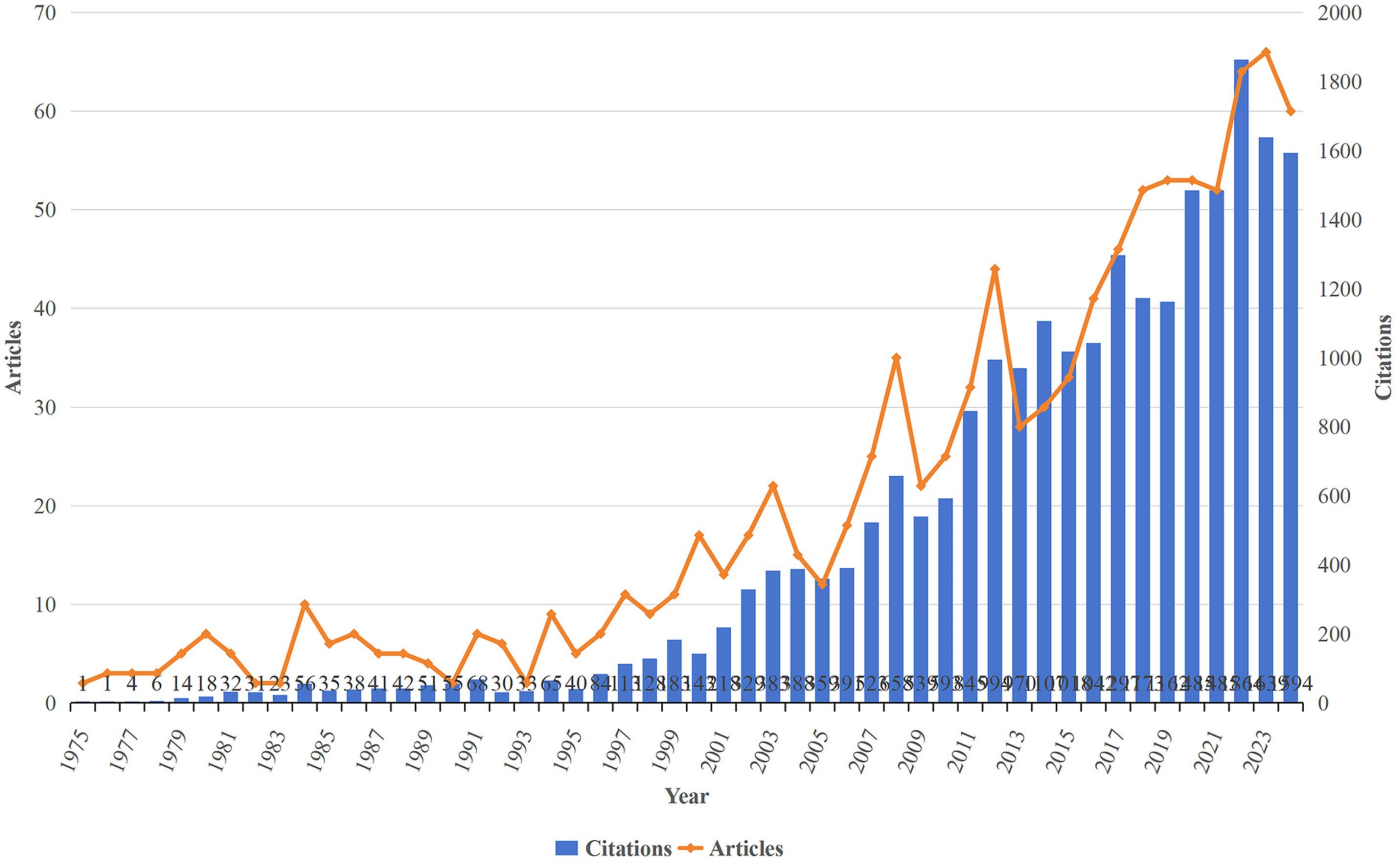
Annual number and the cumulative number of publications.

From January 1, 1975, to October 31, 2024, a total of 1,021 HA-related publications spanning a period of 49 years were retrieved. The study workflow is shown in Figure 1. Figure 2 shows the annual and cumulative publication volumes of HA-related literature. Overall, an increasing trend in the number of HA-related annual publications was noted. During the period from 1975 to 1999, only a handful of studies were published in this field, and after the year 2000, the number of publications generally shows a continuous upward trend. The year with the most publications was 2023, which had 66 published papers. An upward trend in the annual citation frequency of HA-related literature was also noted. The year 2022 was found to have the highest annual citation frequency of the literature at 1864.

### 3.2 Analysis of publication volumes and collaboration by country

An analysis of the number of articles published by countries showed that 68 countries/regions had published in the relevant fields. As seen in Table 1, the country with the highest number of publications was the United States of America (USA), with a total of 213 publications, or 20.9% of the total, in the first place. Spain (*n* = 146, 14.3%) ranked second, the Netherlands (*n* = 88, 8.6%) ranked third, and the other countries included England, Germany, Italy, China, Canada, Sweden, and France. Table 1 also shows the top 10 countries in terms of citations. The three countries contributing the most and having the highest number of citations were the USA (6,973), the Netherlands (3,611), and Spain (2,573). Notably, Sweden ranked 4th in citation counts, whereas China, ranking 7th in publication volume, did not make it into the top 10 for citation counts.

**Table 1:** Top 10 countries/regions in terms of the number of publications and the corresponding frequency of citations.

Countries/regions	publications	Countries/regions	Citations
USA	213	USA	6,973
Spain	146	Netherlands	3,611
Netherlands	88	Sweden	2,573
England	84	Spain	2,526
Germany	81	Italy	2,102
Italy	77	England	1758
China	74	Canada	1,654
Canada	58	Germany	1,624
Sweden	37	India	634
France	37	France	621

**Figure 3:**
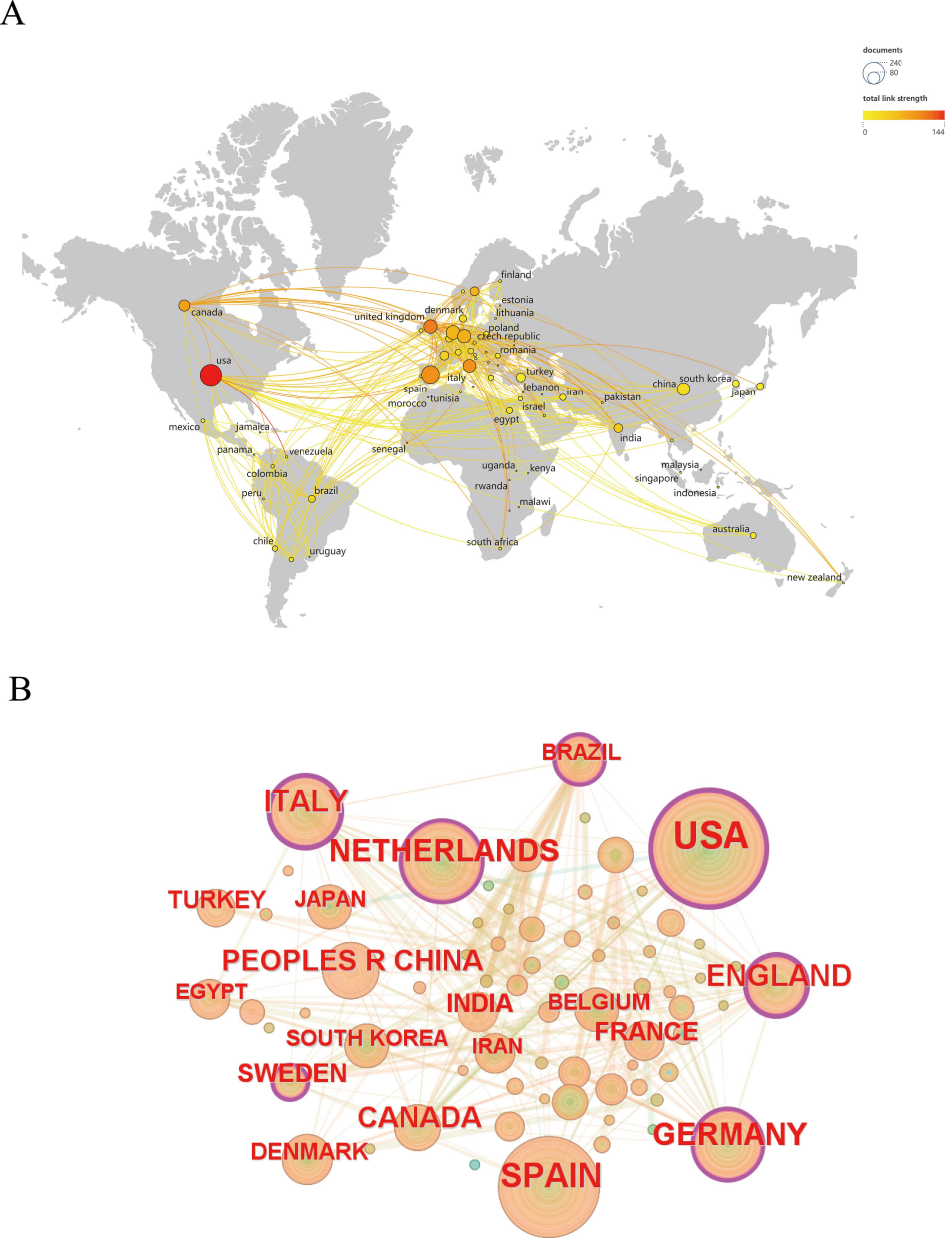
**(A)** Map depicting author collaboration among countries. **(B)** Analytical graph of the cooperation network among countries.

Figure 3A and Supplementary Figure 1 show the cooperation between countries/regions. The main countries with frequent international collaborations were the USA, Spain, and Italy. Among these, the country that collaborated most frequently with others was the USA, which primarily collaborated with Canada, Spain, and China. Notably, connections between countries/regions were mainly concentrated between North America and Europe, with strong links determined between Oceania and Europe, and South America and Europe (Figure 3A). In CiteSpace, if the centrality value of a node exceeds 0.1, it will be marked with a purple circle. These high-centrality nodes occupy a core position in the research network. (Figure 3B). The countries at the center of the international scientific cooperation were the USA, England, Italy, the Netherlands, Germany, Sweden, and Brazil.

### 3.3 Analysis of institutional publications and collaborations

**Figure 4:**
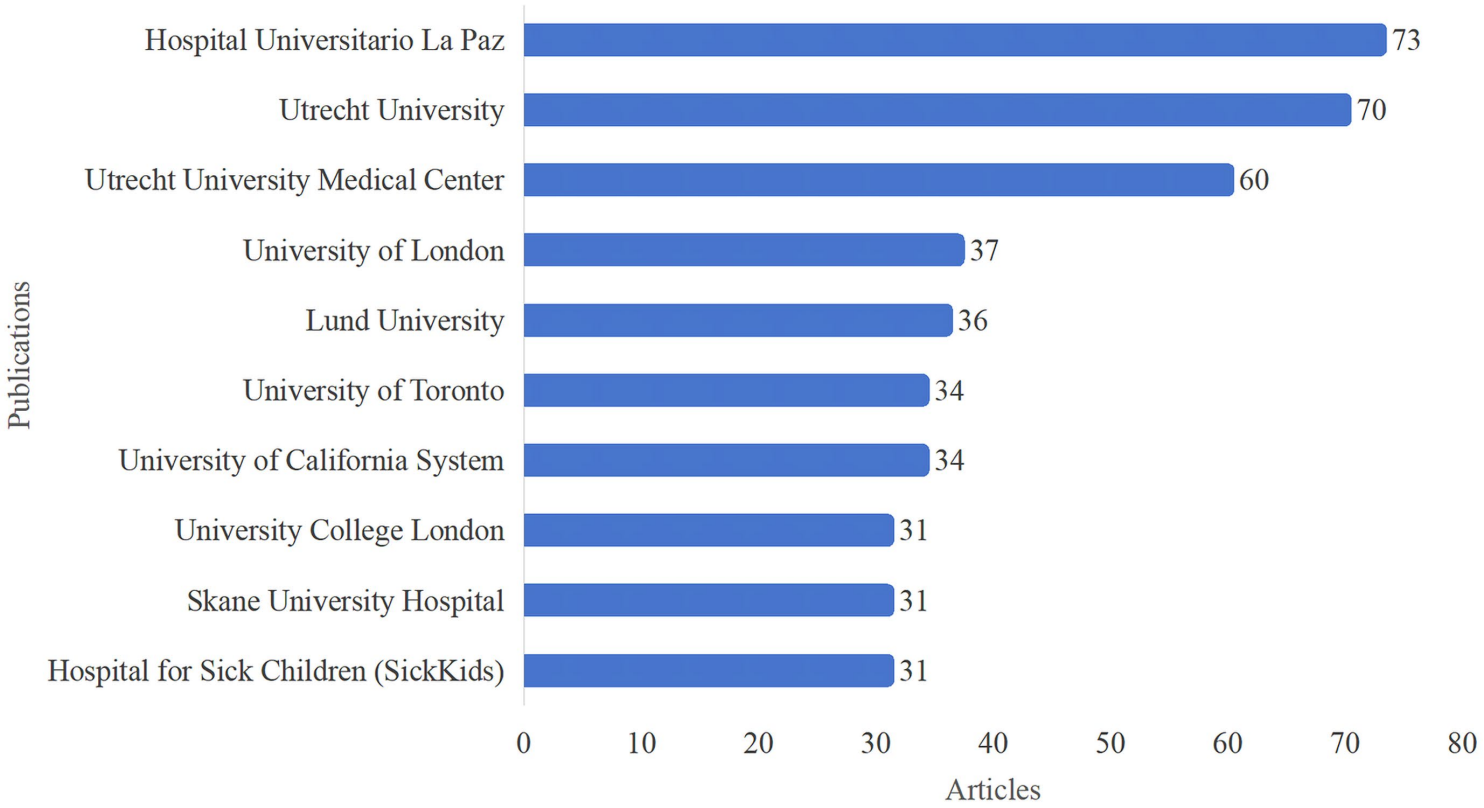
Top 10 institutions with the most publications.

Overall, a total of 1,235 institutions have conducted HA-related research. The top 10 institutions are shown in Figure 4. Hospital Universitario La Paz in Spain had the highest number of publications (73) and ranked first, followed by Utrecht University (70) and Utrecht University Medical Center (60). Among these 10 institutions, two were from the Netherlands, two from Sweden, two from the United Kingdom (UK), and two from Canada; the remaining institutions were from the USA and Spain.

**Figure 5:**
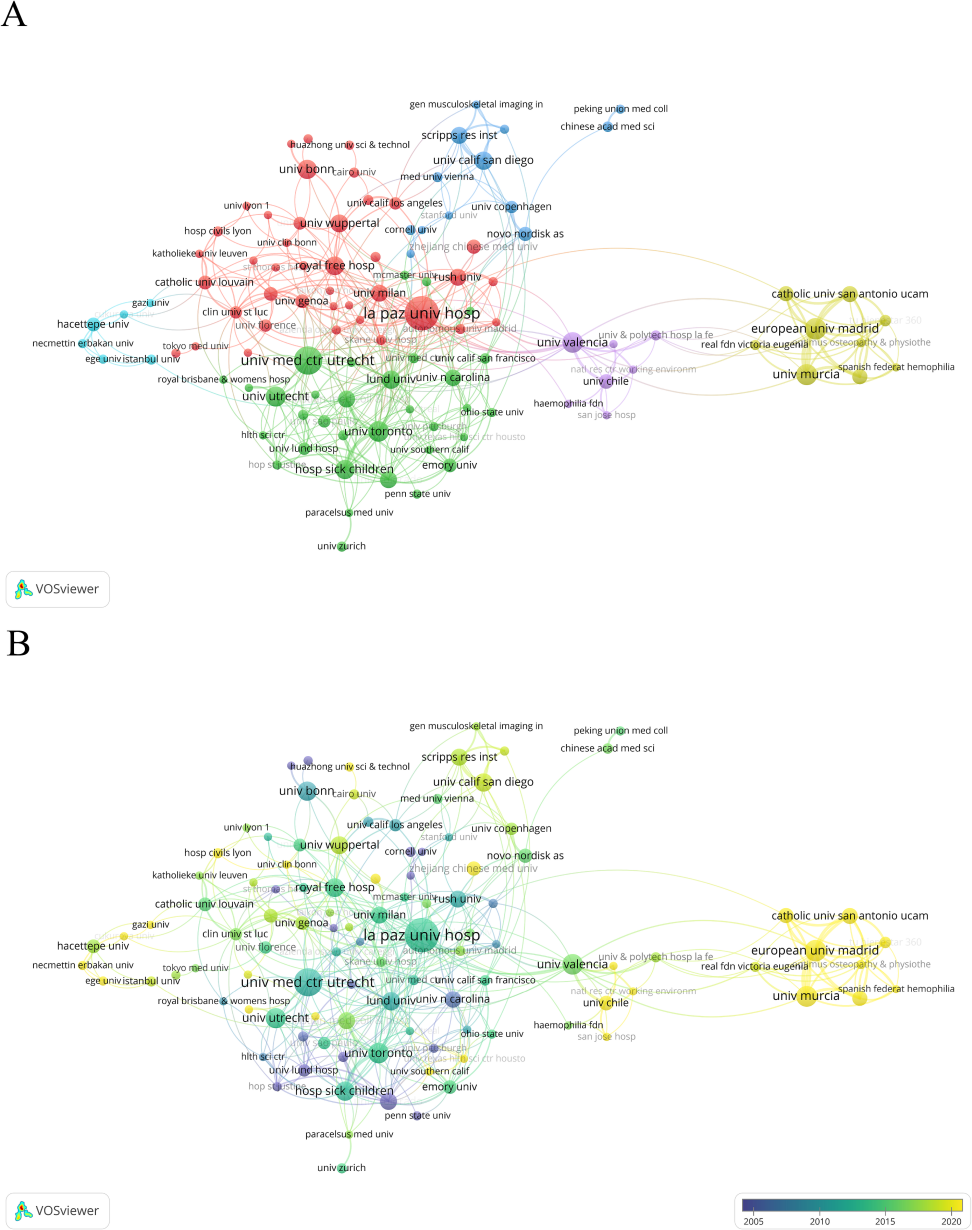
**(A)** Clustering network visualization for institutional co-authorship analysis. **(B)** Time-overlapping visualization for institutional co-authorship analysis.

In addition, a co-authorship analysis of institutions was undertaken to understand the global distribution of HA-related research. Institutions were categorized into six clusters as seen in the clustering network (Figure 5A). The red color scheme indicates the highest number of clustered institutions, containing 40 institutions. Figure 5B shows the average year of publication in the field of HA by institution. Research institutions represented by Skane University Hospital and Cornell University appear to be the early pioneers in this field. Researchers at the European University of Madrid and the University of Murcia have appeared active in the field of HA in recent years.

### 3.4 Analysis of journal paper output and impact

Analysis of journal publications and impact revealed a total of 290 journals to be involved in the publication of the 1,021 documents retrieved in this study. Table 2 lists the top 10 journals and their latest impact factors in order of the number of articles published. HEMOPHILIA from England ranked first with a total of 340 articles. Of these top 10 journals, six were categorized based on Journal Citation Reports (JCRs) Quarter 1 (Q1). Five publishers were from England, three from the USA, and the remaining from Switzerland and Germany.

**Table 2:** Top 10 journals in terms of the number of publications, corresponding impact factor (IF), and JCR quartile.

Rank	Journal	Publications	Country	IF	JCR quartile
1	HEMOPHILIA	340	England	3	Q2
2	BLOOD COAGULATION AND FIBRINOLYSIS	26	USA	1.2	Q4
3	CLINICAL ORTHOPAEDICS AND RELATED RESEARCH	22	USA	4.4	Q1
4	EXPERT REVIEW OF HEMATOLOGY	18	England	2.3	Q2
5	JOURNAL OF CLINICAL MEDICINE	16	Switzerland	3	Q1
6	JOURNAL OF THROMBOSIS AND HAEMOSTASIS	16	England	5.5	Q1
7	THROMBOSIS AND HAEMOSTASIS	13	Germany	5	Q1
8	BMC MUSCULOSKELETAL DISORDERS	11	England	2.2	Q2
9	SEMINARS IN THROMBOSIS AND HEMOSTASIS	11	USA	3.6	Q1
10	BONE AND JOINT SURGERY (former name: JOURNAL OF BONE AND JOINT SURGERY BRITISH VOLUME)	11	England	4.9	Q1

**Figure 6:**
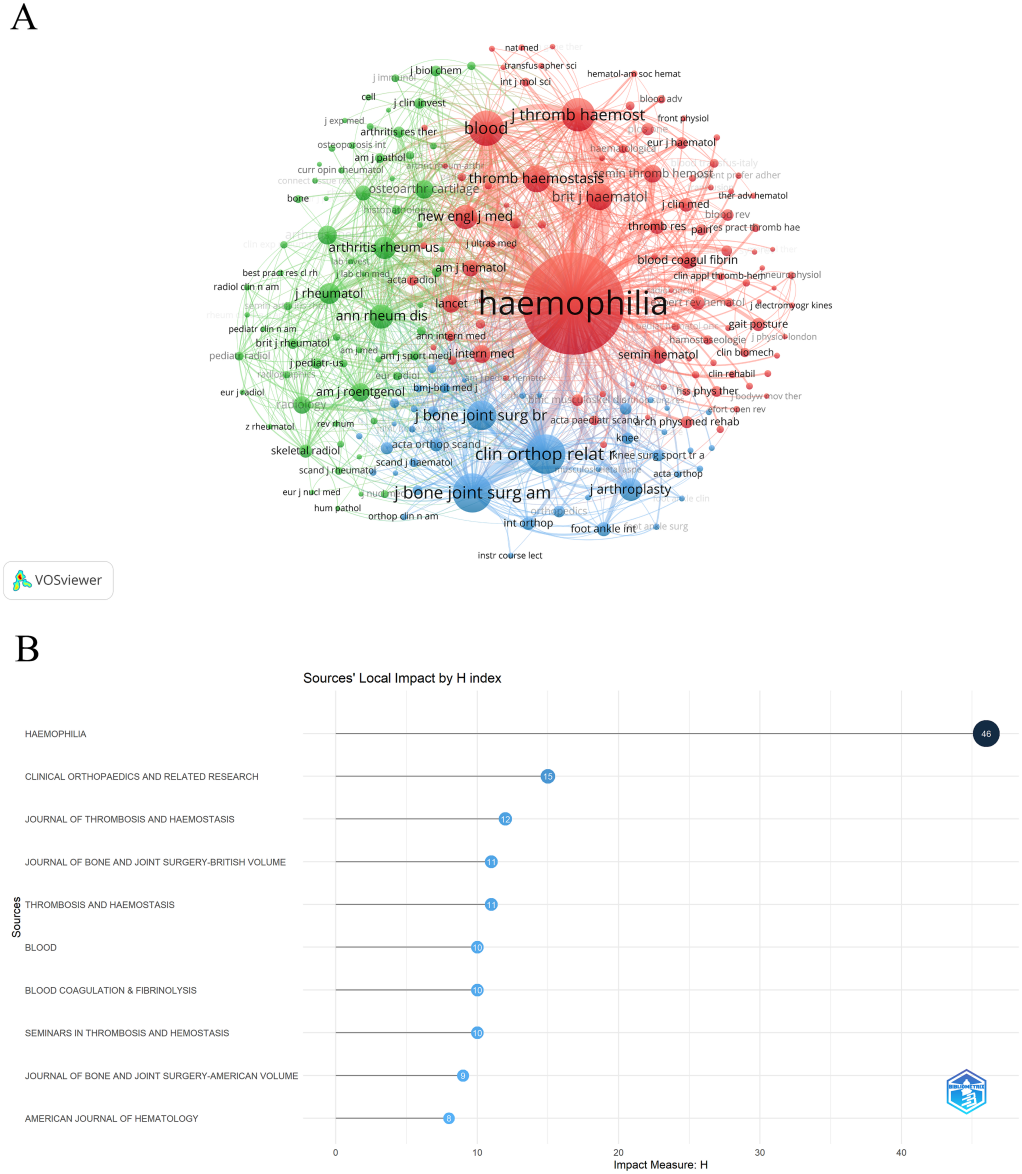
**(A)** Clustering network visualization for journal co-citation analysis. **(B)** Top 10 H-index of 290 journals.

Based on co-citation frequency, these journals were divided into three categories, with their research directions tending toward similarity (Figure 6A). The red cluster focuses on hematology-related fields (HEMOPHILIA, BLOOD, JOURNAL OF THROMBOSIS AND HAEMOSTASIS, etc.); the green cluster centers on immunology (ANNALS OF THE RHEUMATIC DISEASES, ARTHRITIS AND RHEUMATOLOGY, etc.); and the blue cluster mainly indicates journals from the field of orthopedics (CLINICAL ORTHOPAEDICS AND RELATED RESEARCH, JOURNAL OF BONE AND JOINT SURGERY-AMERICAN VOLUME, etc.). Figure 6B shows the H-index of the 290 journals. The journal HEMOPHILIA, which has the highest number of published articles, is also ranked first based on the H-index, indicating the high impact value of the journal in the field of HA.

### 3.5 Analysis of author publications and collaborations

**Figure 7:**
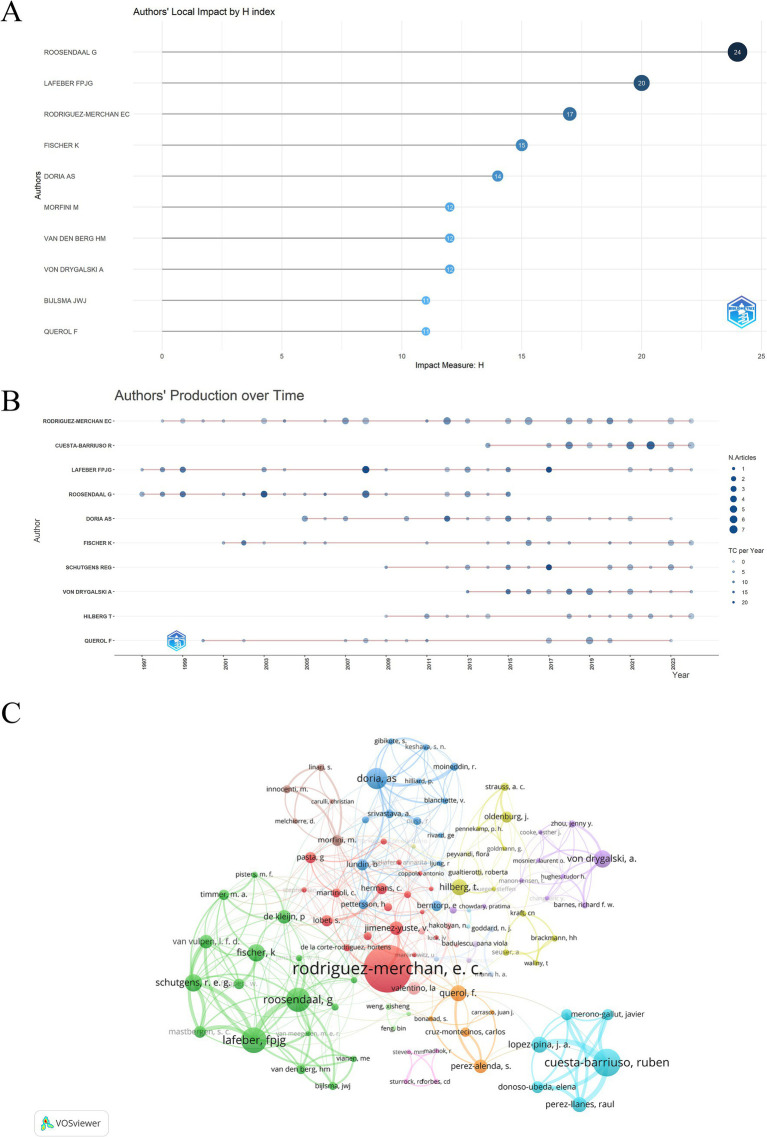
**(A)** Top 10 hemophilic arthropathy domain author H-index. **(B)** Annual number of publications and frequency of citations of high output authors. **(C)** Clustering network visualization for co-authorship analysis among authors.

A total of 3,512 authors participated in the HA study. The author with the most publications was Rodriguez-Merchan EC (Hospital Universitario La Paz, Spain) (54), followed by Cuesta-Barriuso R (University of Oviedo, Spain) (35) and Lafeber FPJG (University Utrecht, Netherlands) (Table 3) (32). Figure 7A illustrates the local impact H-index of the top 10 authors of the papers related to HA. The top three are Roosendaal G (24), Lafeber FPJG (20), and Rodriguez-Merchan EC (17). In addition, Roosendaal G ranked 4th in terms of the number of publications but ranked first in terms of H-index. Similarly, Morfini M, Van den Berg HM, and Bijlsma JWJ ranked among the top 10 in the H-index but were not in the top 10 in the number of articles published. These findings indicate a very high quality of their articles.

**Table 3:** Top 10 authors in terms of the number of publications and corresponding institutions.

Rank	Author	Publications	Institutions
1	Rodriguez-Merchan EC	54	Hospital Universitario La Paz (Spain)
2	Cuesta-Barriuso R	35	University of Oviedo (Spain)
3	Lafeber FPJG	32	University Utrecht (Netherlands)
4	Roosendaal G	30	Utrecht University Medical Center (Netherlands)
5	Doria AS	26	Hospital for Sick Children (Canada)
6	Fischer K	21	Utrecht University Medical Center (Netherlands)
7	Schutgens REG	21	University Utrecht (Netherlands)
8	Von Drygalski A	21	University of California San Diego (USA)
9	Hilberg T	19	University of Wuppertal (Germany)
10	Querol F	19	University of Valencia (Spain)

The annual publication and citation counts for the top 10 authors related to publications are shown in Figure 7B. The size of the data ring is proportional to the number of articles published by the authors. The darker the color of the data ring, the higher the number of citations of the authors’ annual publications. These results show that 9 of the top 10 authors have published in the last 5 years in terms of publications, with Cuesta-Barriuso R being the most active author in the past 5 years. Rodriguez-Merchan EC was found to be the most prolific author who started to focus on this field in 1998 and has since been active. Collaboration between researchers was analyzed using VOSviewer, and the 100 authors were divided into 15 clusters as shown in Figure 7C. Lafeber FPJG, Roosendaal G, Schutgens REG, Fischer K, and others appear to be a part of the same cluster where they have a close collaborative relationship. This cluster, along with the cluster that includes Rodriguez-Merchan EC, Cuesta-Barriuso R, Doria AS, Querol F, Hilberg T, Morfini M, Valentino LA, and others, forms a larger collaborative network. The team led by Von Drygalski A shows frequent collaboration within their own group and hardly any external collaborations. Supplementary Figure 2 shows a network diagram of all cited authors generated using CiteSpace. The top 5 most cited authors are Rodriguez-Merchan EC (401), Manco-Johnson MJ (243), Pettersson H (241), Roosendaal G (225), and Arnold WD (219).

### 3.6 Highly cited reference analysis

**Figure 8:**
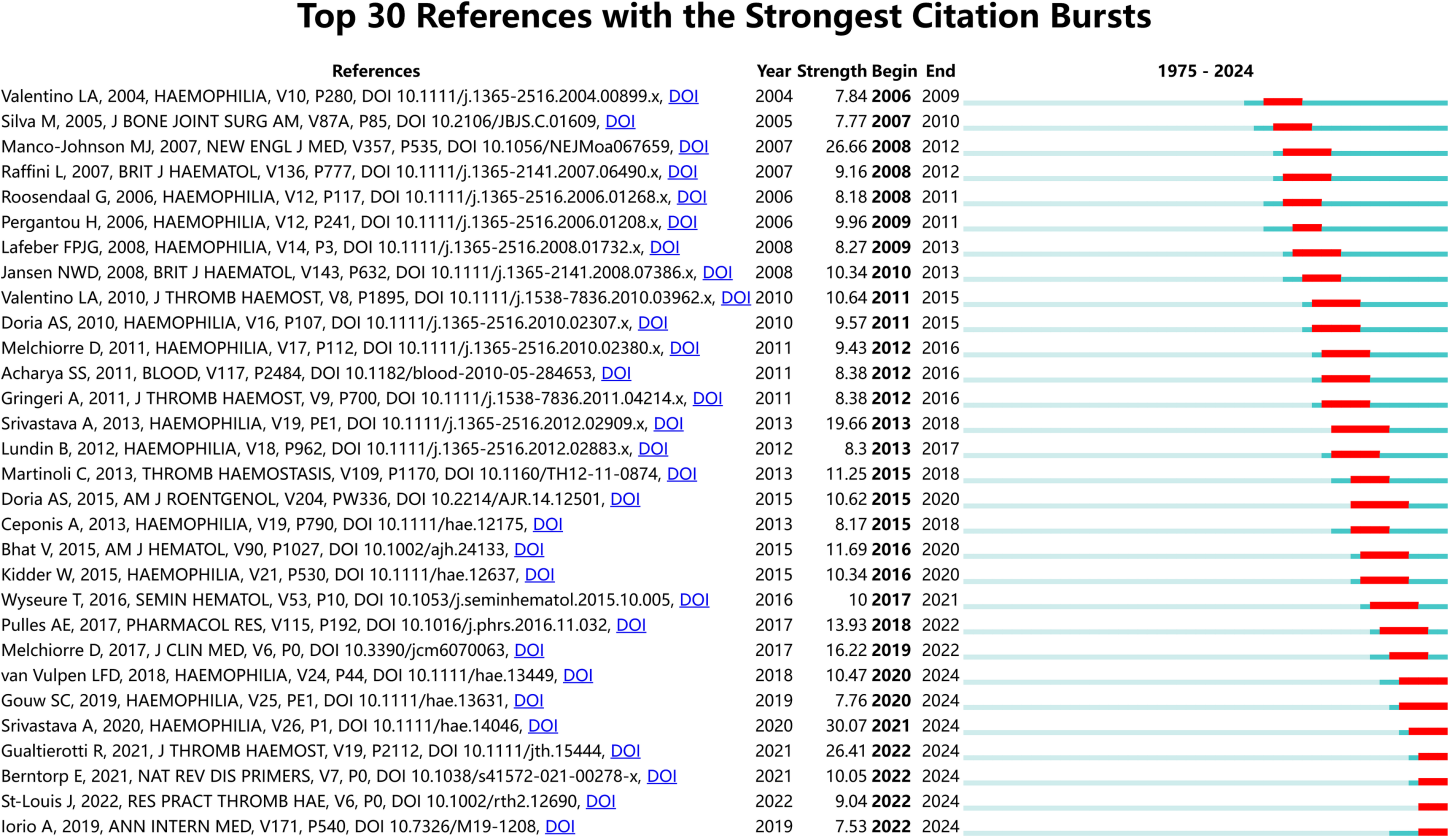
Top 30 references with the strongest citation bursts.

The most cited publications in a field reveal the impact of research in that field. Table 4 shows the top 10 most frequently cited publications. As seen in the list, the articles were published between 1977 and 2013. The most cited article is “Twenty-five years’ experience of prophylactic treatment in severe hemophilia A and B (20)” published in 1992, which focuses on the prophylactic treatment of hemophilic arthritis in children. The 2nd most cited document was published in the JOURNAL OF BONE AND JOINT SURGERY-AMERICAN VOLUME in 1977 titled “Hemophilic arthropathy. Current concepts of pathogenesis and management (21).” Figure 8 shows the top 30 references with the strongest citation bursts. The literature with the strongest citation burst is the article titled “WFH guidelines for the management of hemophilia, 3rd edition (22)”(2021–2024 citation burst = 30.07), written by Srivastava A et al. The article that ranks second is written by Manco-Johnson MJ et al. (citation burst = 26.66), titled “Prophylaxis versus episodic treatment to prevent joint disease in boys with severe hemophilia (23).” In recent years from 2020 to 2024, a total of 7 articles have shown sustained citation bursts, among which the second-highest citation burst can be attributed to “Hemophilic arthropathy: Current knowledge and future perspectives (7)” by Gualtierotti R (citation burst = 26.41).

**Table 4:** Top 10 highly cited articles.

Rank	Article title	Source title	Authors	Year	Cited	DOI
1	Twenty-five years’ experience of prophylactic treatment in severe hemophilia A and B	JOURNAL OF INTERNAL MEDICINE	Nilsson et al.	1992	799	10.1111/j.1365-2796.1992.tb00546.x
2	Hemophilic arthropathy. Current concepts of pathogenesis and management	JOURNAL OF BONE AND JOINT SURGERY-AMERICAN VOLUME	Arnold et al.	1977	487	10.2106/00004623-197759030-00001
3	A radiologic classification of hemophilic arthropathy	CLINICAL ORTHOPAEDICS AND RELATED RESEARCH	Pettersson et al.	1980	405	
4	Hemophilia prophylaxis in young patients - a long-term follow-up	JOURNAL OF INTERNAL MEDICINE	Lofqvist et al.	1997	280	10.1046/j.1365-2796.1997.130135000.x
5	Blood-induced joint disease: the pathophysiology of hemophilic arthropathy	JOURNAL OF THROMBOSIS AND HAEMOSTASIS	Valentino	2010	219	10.1111/j.1538-7836.2010.03962.x
6	The effects of postponing prophylactic treatment on long-term outcome in patients with severe hemophilia	BLOOD	Fischer et al.	2002	210	10.1182/blood.V99.7.2337
7	Pathogenesis of hemophilic arthropathy	HEMOPHILIA	Roosendaal et al.	2006	206	10.1111/j.1365-2516.2006.01268.x
8	Development and definition of a simplified scanning procedure and scoring method for Hemophilia Early Arthropathy Detection with Ultrasound (HEAD-US)	THROMBOSIS AND HAEMOSTASIS	Martinoli et al.	2013	188	10.1160/TH12-11-0874
9	Iron deposits and catabolic properties of synovial tissue from patients with hemophilia	JOURNAL OF BONE AND JOINT SURGERY-BRITISH VOLUME	Roosendaal et al.	1998	175	10.1302/0301-620X.80B3.7807
10	Hemophilic arthropathy	JOURNAL OF THE AMERICAN ACADEMY OF ORTHOPAEDIC SURGEONS	Luck et al.	2004	152	10.5435/00124635-200407000-00004

### 3.7 Keyword analysis

**Figure 9:**
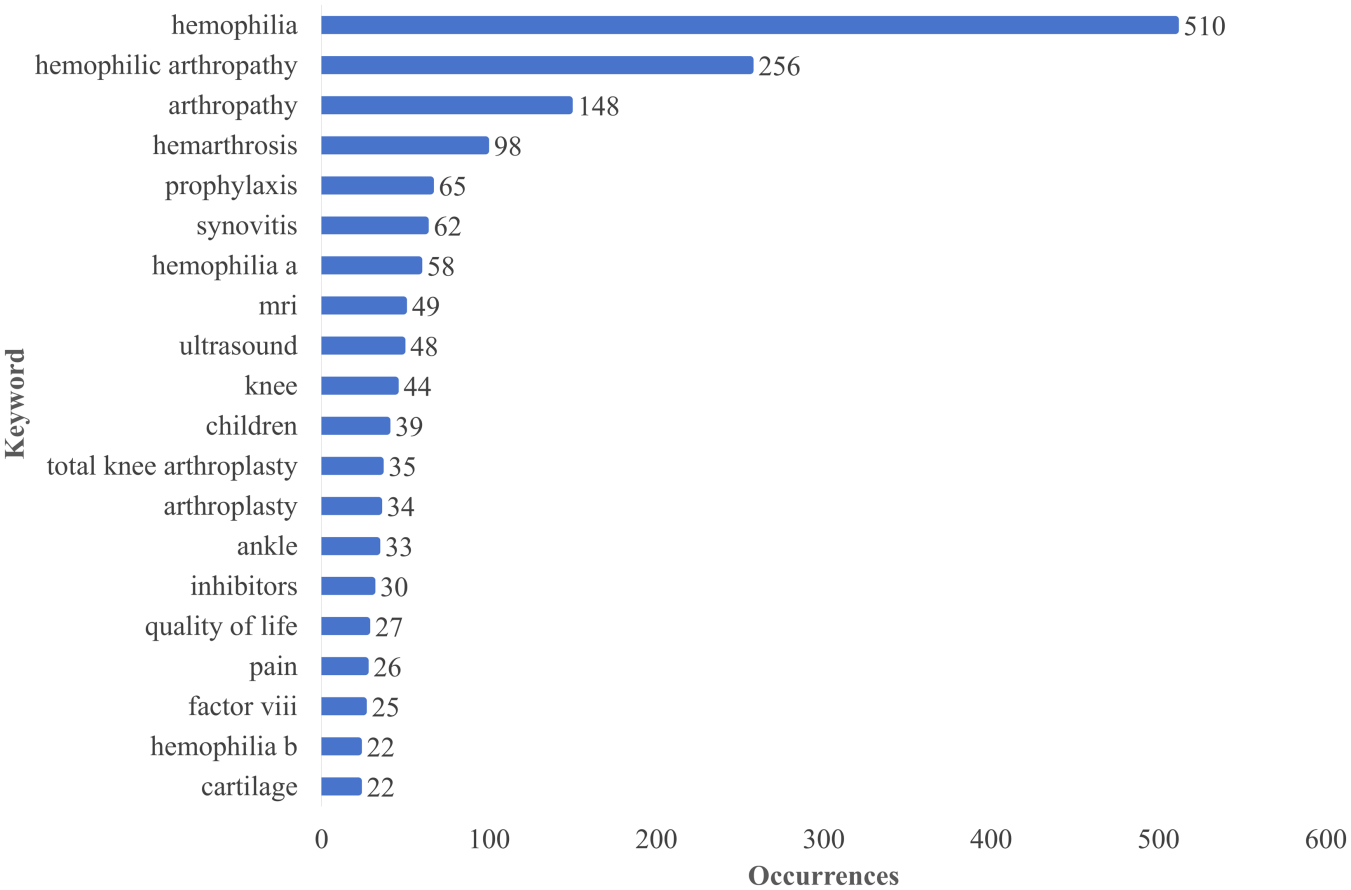
Top 20 most used keywords.

Keywords serve as an overview of the core content of an article. Their analysis can help identify research hotspots in the field understand evolutions in the field and predict future trends. Figure 9 shows the top 20 keywords in the order of frequency. The keyword “hemophilia” was the most common with 510 occurrences followed by “hemophilic arthropathy” (265) “arthropathy” (148) “hemarthrosis” (98) “prophylaxis” (65) and “synovitis” (62). Among the top 20 keywords 2 types of HA namely “knee” (44) and “ankle” (33) appeared.

**Figure 10:**
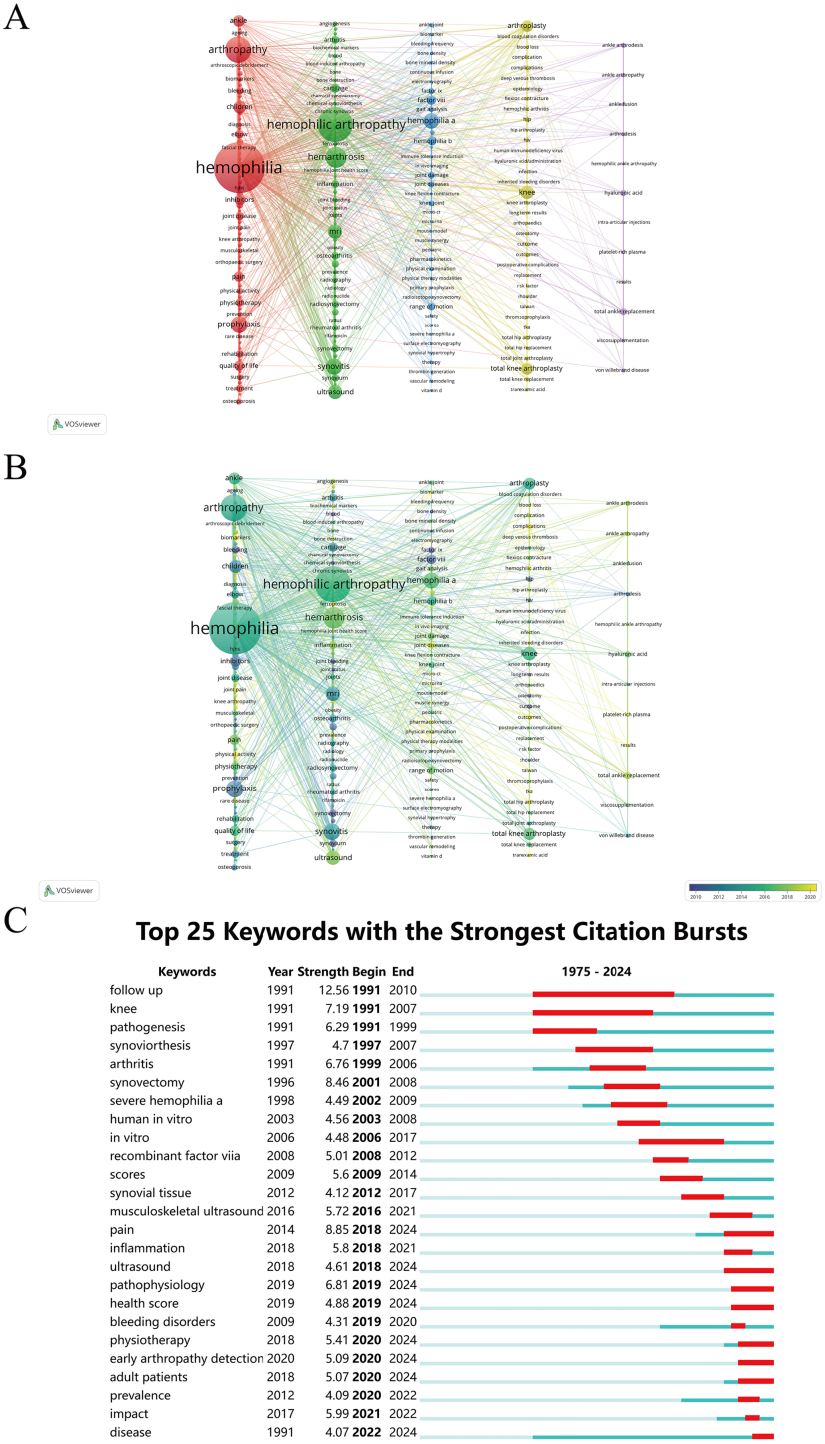
**(A)** Keyword co-occurrence network. **(B)** Keyword co-occurrence plus time-overlapping network. **(C)** Top 25 keywords with the strongest citation bursts.

The co-occurrence network diagram of keywords was visualized using VOSviewer (Figure 10A). The keywords were clustered according to the research direction and roughly divided into the following five categories: keywords clustered in red are related to the well-being of patients with HA (pain, quality of life, etc.), preventive treatments, and management (prophylaxis, children, inhibitors, physiotherapy, rehabilitation, exercise, etc.). Keywords in the green cluster are related to the pathogenesis of HA (hemarthrosis, synovitis, inflammation, cartilage, etc.), diagnosis (ultrasound, MRI, etc.). Keywords in the blue cluster are related to HA typing (hemophilia A, factor VIII, hemophilia B, factor IX, etc.), functional assessment (range of motion, gait analysis, hemophilia joint health score, etc.). Keywords in the yellow cluster are related to HA replacement surgery (knee, hyaluronic acid, arthroplasty, hip, complications, etc.). Keywords in the purple clusters are related to hemophilic ankle arthritis treatment (total ankle replacement, synoviorthesis, ankle arthrodesis, etc.).

Figure 10B shows the temporal overlapping analysis network of the co-occurring keywords. Early studies primarily focused on areas such as factor IX, synoviorthesis, and synovectomy. In contrast, in recent years, topics such as joint diseases, detection of early arthropathy, kinesiophobia, and ferroptosis have received increasing attention.

Figure 10C presents the top 25 keywords with the strongest citation bursts. The keywords “follow-up” (outbreak duration 1991 to 2010, 19 years) and “knee” (1991 to 2007, 16 years) have received the most sustained attention over time. In addition, notable keywords for 2020 to 2024 were “physiotherapy,” “adult patients,” “early arthropathy detection,” and “disease,” suggesting that these keywords represent hot research topics in recent years and the near future.

### 3.8 Analyzing different HA joint types

The types of joints most frequently involved in the onset of HA topics are shown in Table 5. The total number of articles in which HA morbidity involved different joint types was counted, and the highest number of articles (80) were related to the knee. The other common joints included the ankle (61), elbow (22), hip (12), and shoulder (4).

**Table 5:** Top 5 joint types.

Joint type	Number
Knee	80
Ankle	61
Elbow	22
Hip	12
Shoulder	4
Total	179

## 4 Discussion

### 4.1 Status and trends of global research

Bibliometric analysis was used in this study to analyze the literature in the field of HA from 1975 to 2024, and the earliest article was found to be published in 1975. The number of annual publications indicates a continuous upward trend in the number of publications overall after 2000. In 2018–2024, the number of annual publications was more than 50 articles. These findings indicate that HA is likely to remain in an active and robust phase in the near future. The potential reason underlying this phenomenon may be that as the development of modern medicine provides patients with more precise, effective, and personalized multistage treatments, an increasing number of institutions are devoting more attention and support to field of HA, leading to a high growth in research rate in recent years.

Results from the current analysis indicate that the USA dominates the field with the largest number of publications and an important position in international cooperation. USA ranks first in terms of publication volume and citation frequency, whereas the Netherlands ranks third in terms of publication volume and second in terms of citation frequency. This study demonstrated that the USA, UK, Italy, the Netherlands, Germany, Sweden, and Brazil are the primary countries for HA-related research. Close communication and cooperation were also determined to be possible in the Asian region where their respective regions lack cooperation. In addition to countries with a strong influence in the field such as the USA, the Netherlands, and Spain, some European countries that play an important role are also prioritized for cooperation. Collectively, countries in a leading position can strengthen their cooperation with other countries to further promote the overall development of the field of HA.

Among the top 10 institutions in terms of publications, two are from the Netherlands, two are from Sweden, two are from the UK, two are from Canada, and the remaining institutions are from the USA and Spain. Despite USA ranking the first in terms of publications, only one of the top 10 institutions is from the USA. Germany ranks fifth in national publication output, but none of the institutions in the top 10 are from Germany. In recent years, the European University of Madrid and the University of Murcia have been particularly active.

This study revealed that the journal HEMOPHILIA has the highest number of publications, ranking first both in terms of co-citation frequency and H-index and being a core journal in the field of HA. Among the top 10 journals, four publishers are from the USA and four are from England, with JCR Q1 journals accounting for 60%. In addition, journals from neither Spain nor the Netherlands are in the top 10 rankings in this field despite their significant contribution to the field. This finding shows that both these countries have adequate capacity to create journals that have an international impact. It is noteworthy that among the top 10 journals in terms of frequency, three are related to orthopedics (CLINICAL ORTHOPAEDICS AND RELATED RESEARCH, BMC MUSCULOSKELETAL DISORDERS, BONE AND JOINT SURGERY).

This study found that Roosendaal G is the top-ranking author in terms of the H-index. Roosendaal G is from Utrecht University Medical Center, the Netherlands. In 1998, Roosendaal et al. (24) published the article “Iron deposits and catabolic properties of synovial tissue from patients with hemophilia” in the JOURNAL OF BONE AND JOINT SURGERY-BRITISH VOLUME, verifying that localized synovial iron deposits promote the expression of synovial inflammatory factors and increase the catabolic activity of synovial tissues in patients with HA. Moreover, Roosendaal et al. (25) published “Pathogenesis of hemophilic arthropathy” in 2006, an article that reviewed the pathogenesis of HA and significantly contributed to the study of the pathogenesis of HA. Rodriguez-Merchan EC, being the author with the highest number of publications and citations, and ranking third in the H-index, is recognized to be an authoritative figure in the field of HA. This author mainly engages in research related to the diagnosis of HA, nonsurgical therapies, and surgical treatments for the condition (26–28). The team led by Cuesta-Barriuso R has been the most active in recent years, focusing on improving the quality of life of patients with HA and on the evaluation and treatment of disorders of the musculoskeletal system as well as pain management, exercise therapy, and rehabilitation training (29–32).

### 4.2 Current research hotspots

Keywords represent the core content of the research and high-frequency keywords indicate current research trends in the field that help understand the cutting-edge research related to this field and HA-related research hotspots. The results of our study show that the main keywords in the existing studies in this field include “hemophilia,” “hemophilic arthropathy,” “arthropathy,” “hemarthrosis,” “prophylaxis,” “synovitis,” “hemophilia a,” “MRI,” and “ultrasound” (Figure 9) and that these keywords are mainly related to the pathogenesis prophylaxis and diagnosis of HA suggesting them to be popular topics in hemophilic arthropathy. The co-occurrence network diagram of keywords and analysis of the literature that has been highly cited show that the research hotspots in the field of HA mainly involve HA pathogenesis diagnosis preventive treatment surgical treatment and patients’ quality of life.

#### 4.2.1 Pathogenesis of HA

Recurrent intra-articular bleeding is a predominant cause of HA. Recurrent joint bleeding leads to two major outcomes, namely, synovitis and osteochondral damage, which interact and lead to the development of arthropathy. As earliest complication of joint hemorrhage is synovitis, which is characterized by synovial hypertrophy, inflammatory cell recruitment, and a high degree of neovascularization. Repeated blood accumulation in the joints leads to the accumulation of ferritin in the synovium and activation of the nuclear factor-kappa B signaling pathway, resulting in an inflammatory response (33–35). Iron can induce synovial cell proliferation and inhibit synovial cell apoptosis, contributing to synovial hypertrophy (36, 37). Inflammation and synovial hypertrophy can promote vascular endothelial growth factor (VEGF) expression and synovial angiogenesis, which, in turn, increases the risk of further bleeding (4, 38). The interaction between iron, inflammation, hypertrophied synovium, and neovascularization forms a vicious circle. Hyperplastic synovium can also lead to the production of inflammatory cytokines, collagenolytic enzymes, fibrinolytic enzymes, and matrix metalloproteinases, all of which mediate cartilage damage (39, 40).

Blood can cause direct damage to the cartilage because hydroxyl radical formation can lead to chondrocyte apoptosis and impaired renewal of the extracellular matrix (41, 42). Alterations in local bone homeostasis due to the production of pro-inflammatory cytokines and proteases create an imbalance in the RANKL/RANK/OPG pathways, ultimately leading to increased bone resorption and subchondral damage (43, 44). Arthrofibrosis is the most serious disabling complication in patients with HA that is likely related to the production of connective tissue growth factor by synoviocytes (45). The pathogenesis of HA is a highly complex pathophysiological process and its mechanism has not been fully elucidated. Possible mechanisms include ferritin deposition and synovial inflammation, as well as a high extent of neovascularization, cartilage destruction, subchondral bone destruction, and arthrofibrosis interactions that promote disease progression. However, the exact pathogenesis of HA is unclear, making it a much sought-after research hotspot.

#### 4.2.2 Diagnosis of HA

The current diagnosis of HA is mainly via imaging, which commonly includes MRI, ultrasonography, and X-ray. MRI is the diagnostic tool of choice for HA, as it can effectively identify abnormal manifestations such as early joint damage, synovitis, subchondral cysts, cartilage loss, and ferritin-containing deposits. Furthermore, it can easily identify early joint lesions and lesion progression in individuals with hemophilia (46, 47). However, this modality is expensive and time consuming. X-ray has low sensitivity in early arthropathy but is useful in assessing advanced structural joint damage and determining the extent of osteoporosis (46). With the continuous development of ultrasonography in the field of HA, examination techniques using this modality have been successful in detecting and quantifying activity in arthritic conditions as well as in identifying degenerative changes in the joints, thereby providing an important basis for the development of therapeutic and rehabilitation programs (48). Ultrasonography can also rapidly and accurately identify joint hemorrhage and cartilage lesions (10, 49, 50) and is more sensitive than MRI in differentiating between hemorrhagic and nonhemorrhagic effusions (51). In 2013, Martinoli et al. (52) developed an ultrasound-detection score for early hemophilic arthropathy in patients with hemophilia, which provides a standardized assessment tool for the use of ultrasound for the diagnosis of early HA. In children, due to the specificity of joints, which are characterized by epiphyses that are not fully ossified, ultrasound is ineffective in distinguishing between cartilage and epiphyseal demarcation. Therefore, ultrasonography is a modality that has limited applications for the early diagnosis of hemophilic osteoarthropathy in children. Serum and synovial biomarkers are also an important research direction. The abnormal expression of biomarkers such as COMP, VEGF, RANKL, and OPG has been observed in patients with HA (53). However, the clinical value of these markers remains unclear. Serum and synovial biomarkers that are highly specific for HA are still lacking. Integrating clinical, imaging, and serum markers for the diagnosis and treatment of HA to assess the arthropathy grading of patients for the development of personalized treatment is the best approach to preserve joint function.

#### 4.2.3 Prophylaxis for HA

Preventive therapy and early intervention are the best treatment options for osteoarthropathy in patients with hemophilia (54). In 1992, Nilsson et al. (20) conducted a clinical study for early and effective continuous prophylaxis in children with hemophilia aged 3–7 years and found that early and effective prophylaxis and preventing the occurrence of hemorrhage and HA when the concentration of factor VIII coagulant activity (VIII:C) or factor VIII coagulant activity (IX:C) falls to <1% of normal levels may serve as an important clinical rationale to shift from emergency treatment to regular prophylactic injections after bleeding events. VIII:C or IX:C concentrations <1% of normal levels may prevent bleeding events and the occurrence of HA. Our study presents an important clinical rationale for changing the coagulation factor supplementation from an emergency treatment following a bleeding event to a regular prophylactic injection, accounting for a landmark in the field of HA prophylaxis. In 2002, Fischer et al. (55) quantified the increase in long-term arthropathy due to delayed prophylaxis. Their findings indicated that most patients with hemophilia experience their first joint bleed after the age of two. Through a 20-year follow-up, they discovered that the longer the period between a joint bleed and the initiation of prophylaxis in patients with hemophilia, the higher the risk of developing arthropathy. Early prophylaxis can reduce the incidence of HA and improve the quality of life as well as the physical and mental health of patients with hemophilia. The aim of prophylaxis is to prevent joint hemorrhage, which is the most typical and common type of bleeding in patients with severe hemophilia, and to work toward preventing the long-term adverse effects of hemorrhage on the structure and function of joints, which can lead to HA (25, 56). Prophylactic approaches include clotting factor concentrates, physical therapy and exercise, education and training, and the use of hemostatic drugs to reduce joint damage and maintain mobility (22). These clinical and societal benefits have been clearly described based on relevant studies in the field of HA, indicating that the sooner the prophylaxis is initiated, the better the outcome of joint status of patients. With the development of biotechnology, treatments for HA in combination with nonalternative treatments, such as subcutaneous emicizumab, can improve patient compliance and offer better joint protection (7). The extent of joint bleeding can be reduced by individualized and effective prophylactic approaches; however, evidence suggests that this approach does not address the root causes of synovial hyperplasia and subclinical bleeding (5, 57). Therefore, the development of individualized and effective prevention programs is currently an important research topic. Moreover, the identification of effective approaches to prevent joint bleeding in patients with hemophilia and prolongation of the duration of efficacy to prevent HA is a current research trend (58, 59).

#### 4.2.4 Improved quality of life for patients with HA

Joint pain, joint deformity, and joint dysfunction are the primary symptoms of HA that seriously affect the quality of life of patients and impose a considerable socioeconomic burden (60, 61). Therefore, improving the quality of life of patients with HA and their rehabilitation continues to remain a research hotspot. In 2006, the Physical Therapy Working Group of the International Prevention Research Collaboration published the HJHS scale, which provides a standardized quantitative index to assess joint function and the rehabilitation of joint function in patients with HA (62). This scale was originally designed to determine the extent of mild joint damage in patients with HA aged 4–18 years. This highly reliable index that relies on factor prophylaxis is suitable for children and patients with hemophilia (63, 64). In a multicenter international study, St Louis et al. (65) validated the use of the HJHS scale in adult patients with hemophilia and arthropathy.

Pain in patients with HA is categorized into acute and chronic pain (66, 67). Pain management in patients with hemophilic osteoarthropathy follows a multimodal and multidisciplinary strategy (68). Acute pain in patients with HA is usually caused by acute bleeding, which can usually be arrested by clotting factor–replacement therapy (22) combined with conventional means such as braking, joint protection, ice packs, compression bandages, and elevation of the affected limb (69). Arthrocentesis can help improve joint function in patients with massive intra-articular hemorrhage in the acute phase, enabling them to commence rehabilitation exercises sooner (70). Arterial embolization for hemostasis is a viable option for patients experiencing recurrent bleeding (71). Treatments such as local therapy, physical therapy, exercise therapy, and analgesics can be used for patients with HA who experience chronic pain (58, 72) to improve their joint function and quality of life. Local treatments mainly include intra-articular administration of hyaluronic acid (73) and platelet-rich plasma arthrocentesis (74). Physical therapy can effectively reduce inflammation, loosen adhesions, reduce muscle atrophy, and relieve pain (75). The commonly used physical therapies for HA include transcutaneous electrical stimulation neurotherapy (76), neuromuscular electrical stimulation (77), low-level laser therapy (78), pulsed electromagnetic field (79), acupuncture (80), kinesiologic taping (81), and fascial therapy (82). Exercise therapy for patients with HA includes muscle strength training, endurance training, proprioceptive training, and balance training, all of which are beneficial in restoring the range of motion of joints as well as muscle strength, endurance, proprioceptive and balance coordination functions, and joint stability (83, 84). Cognitive-behavioral therapy can considerably improve the quality of life of patients with HA (85). Acetaminophen/paracetamol is currently the drug of choice to treat mild-to-moderate acute or chronic joint pain in patients with hemophilia (22). Other drugs include selective cyclooxygenase-2 inhibitors and opioids (72). Chronic and recurrent pain affects the psychological state of patients with HA experiencing neuropathic pain, and anticonvulsant and antidepressant medications are available to restore their mental health (86). Pain and joint dysfunction can seriously affect the quality of life of patients with HA. Therefore, the development of individualized and effective pain-management programs and rehabilitation training programs via multidisciplinary and multimodal approaches continue to remain a much sought-after research hotspot.

#### 4.2.5 Surgical treatment for HA

The main surgical treatment modalities for synovitis in patients with HA are major radioisotope synovectomy (87), chemical synovectomy (88), arthroscopic synovectomy (89), and open synovectomy (90). Radioisotope synovectomy and chemical synovectomy are the treatments of choice for synovitis, and arthroscopic synovectomy can be resorted to when noninvasive treatments are not very effective. Patients develop severe joint deformities accompanied by intolerable severe pain with the progression of HA, and arthroplasty is used as the treatment of choice (91). Studies suggest that patients with hemophilic ankle arthropathy can be better treated with ankle fusion than with ankle arthroplasty (92). Periarticular osteotomy is also a good option for young patients having HA with mild arthropathy to correct the anatomical lines of force of the joints and delay arthroplasty (93, 94). Arthroplasty can maximize the improvement of joint function and quality of life of affected patients; however, one should be mindful of orthopedic surgery–related complications. Therefore, developing individualized surgical treatment options to improve the quality of life of patients is a research hotspot.

### 4.3 Future research trends

The keyword “time-series overlap analysis” refers to the sampling of data in a time-series analysis where there is an overlap between the sampling windows. This approach improves data utilization, thereby reducing information loss and improving analytical accuracy. In this study, the analysis of keyword temporal overlap showed that the findings of “adult patients,” “early arthropathy detection,” “acute hemarthrosis,” “kinesiophobia,” and “ferroptosis” were indicative of recent research hotspots. Citation Burst Analysis in CiteSpace analyzes references and keywords that have significant changes over a specific period of time, effectively providing information on recent research hotspots and future research directions. The keywords used in this study were “physiotherapy,” “adult patients,” and “early arthropathy detection,” with the most recent occurrence up to the year 2024. Some of these keywords also appeared in the temporal overlap analysis. In terms of references, there are seven cited publications since 2020 and the burst continues until 2024. Four of these articles were reviews of HA, whereas two articles focused on the health scores of patients with HA. Three of the four studies reviewed the pathogenesis, diagnostic imaging, prophylaxis, rehabilitation, and surgical treatment options for HA (7, 35, 95), whereas one article established HA-treatment guidelines (22). In addition, one article focused on the HJHS rehabilitation score for HA (65). Our findings indicate the reference hotspots to be consistent with the keyword hotspots. Future research hotspots in this field could be for the following 3 areas: (1) Diagnostic imaging and serum indicators with clinical utility for the early diagnosis of HA, especially for the pediatric population; (2) Development of individualized plans to treat HA using multiprotocol and multidisciplinary approaches to improve the quality of life of patients; (3) Elucidation of the pathogenesis of deep HA, such as iron death (96).

Among the different joint types involved in HAs, knee and ankle-related studies ranked the top two, with each topic having more than 60 publications. It could therefore be inferred that knee- and ankle-related studies had the most information related to HA.

## 5 Study limitations

Our study is the first to use bibliometrics and visualization to analyze WOSCC studies related to the field of HA from January 1, 1975, to October 31, 2024. However, it is important to acknowledge some limitations of our study.

First, as this study is a bibliometric analysis, the collection and processing of data were highly software dependent. Although this analysis cannot completely replace a systematic search, it helps with the comprehensive analysis of a large amount of data. Second, the data in this study were solely sourced from English-language documents in the WOSCC, which may lead to potential omissions of some literature data. Therefore, there is a possibility of some valuable data being missed, leading to biased results. However, as the English-language literature in the WOSCC database covers most of the existing studies, we believe that an omission, if any, will not significantly affect our overall findings. Lastly, due to the time lag with respect to citation impact, the impact of some of the recently published high-quality studies may have been underestimated, thereby warranting attention and updating in future studies. Nevertheless, the findings from the current study will help researchers understand the current trends, hotspots, and frontiers in the field of HA.

## 6 Conclusion

This study quantified HA research using the WOSCC database and employing bibliometrics and visualization analysis. Our findings indicate that the field of HA is receiving increasing attention from researchers. The top three countries with the highest number of publications are the USA, Spain, and the Netherlands. HEMOPHILIA was determined to be the most influential journal based on the analysis of the combined number of publications and citations. Rodriguez-Merchan EC was found to be the most authoritative author in the field of HA. Annual publication data reveal an increase in the number of publications in HA-related fields since 2000, a peak in the numbers in 2023, and stabilization with a general upward trend. Based on the co-occurrence network diagram of keywords and the analysis of the literature that was highly cited, five main research directions in the field of HA and the research trends they represent are summarized in this study. The areas that warrant attention in future studies are as follows: (1) Determination of the in-depth mechanism of the pathogenesis of HA; (2) Studies on diagnostic imaging and clinically useful serum indices for the early diagnosis of HA, especially for the pediatric population; (3) Development of individualized treatment plans for HA to improve the quality of life of patients using a multiprotocol, multidisciplinary approach. Overall, our study analyzed current research trends and hotspots in HA-related fields in a timely manner, providing data for researchers to conduct accurate and in-depth studies in HA-related fields for the development of this field and laying the foundation for future research.

## Data Availability

The raw data supporting the conclusions of this article will be made available by the authors, without undue reservation.

## Footnotes

^1^ https://bibliometric.com/

## Supplementary material

SUPPLEMENTARY FIGURE 1 Pie chart representing the quantitative relationship of cooperation between countries.

SUPPLEMENTARY FIGURE 2 Map of cited authors in hemophilic arthropathy.
