# Supplementary figures and images for: Global trends in hemophilic arthropathy research: a bibliometric and visualization analysis
Source: Front Med (Lausanne). 2025 Apr 16;12:1556906. doi: 10.3389/fmed.2025.1556906 (PMC12041055; doi:10.3389/fmed.2025.1556906)

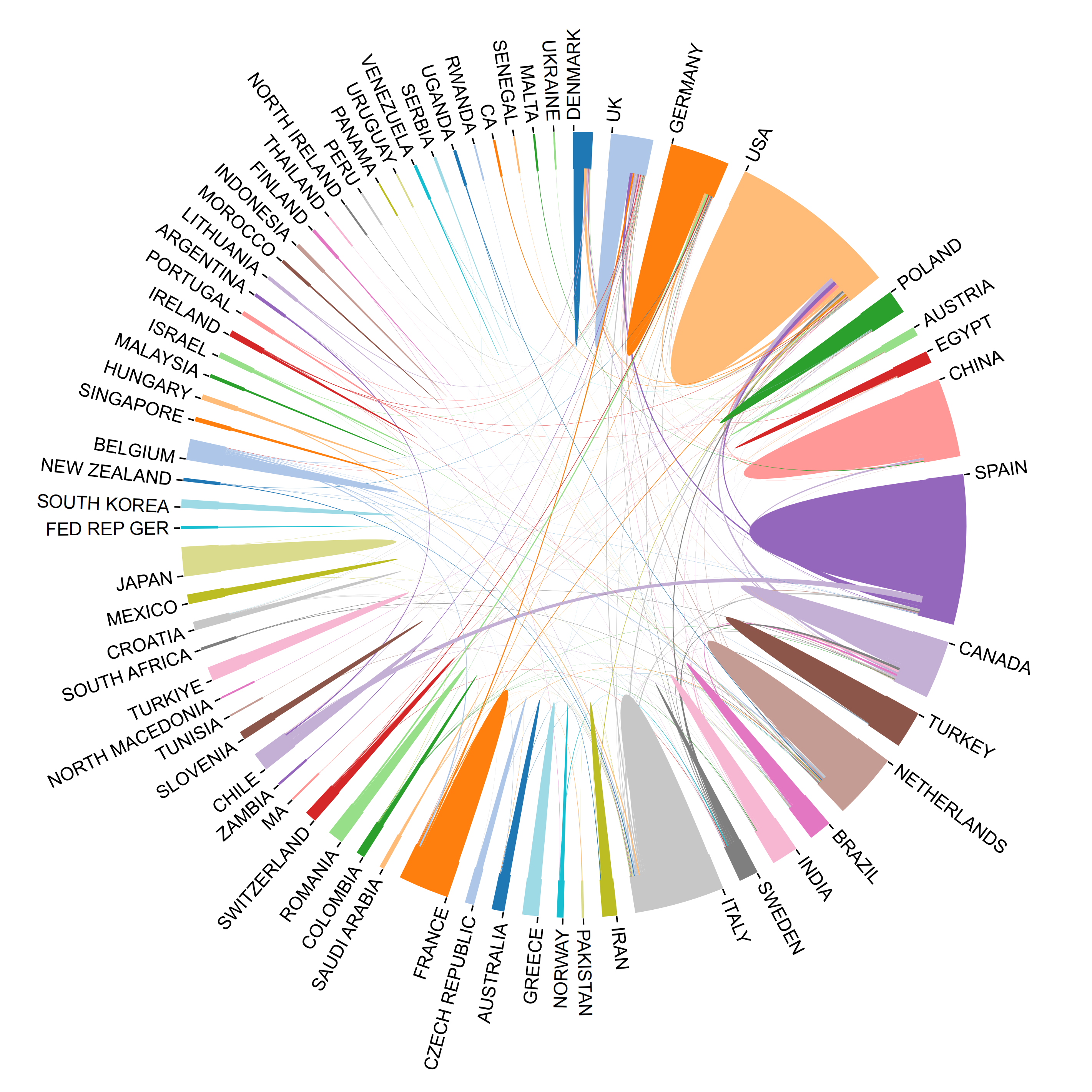

Supplement: SUPPLEMENTARY FIGURE 1 — Pie chart representing the quantitative relationship of cooperation between countries. [file Image_1.TIF]

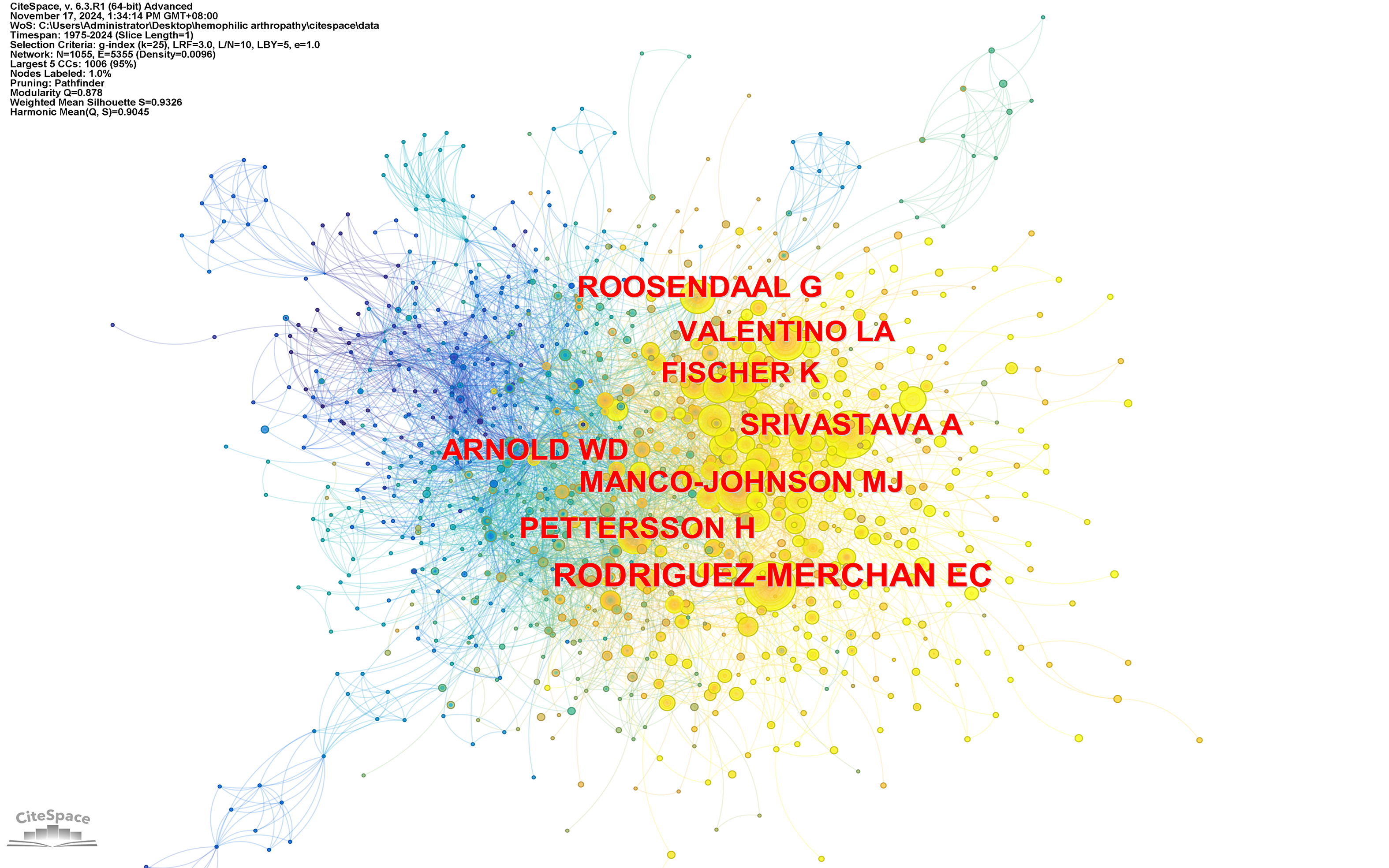

Supplement: SUPPLEMENTARY FIGURE 2 — Map of cited authors in hemophilic arthropathy. [file Image_2.TIF]
