# Supplementary material for: Global trends in hemophilic arthropathy research: a bibliometric and visualization analysis
Source: Front Med (Lausanne). 2025 Apr 16;12:1556906. doi: 10.3389/fmed.2025.1556906 (PMC12041055; doi:10.3389/fmed.2025.1556906)
Supplement: Supplementary file 3 [file Table_1.DOCX]

**Analytical workflow for evaluating different HA joint types**

This study was based on 1,021 hemophilic arthropathy (HA)-related articles retrieved from the Web of Science Core Collection (WOSCC) database. The research dataset was constructed using EndNote X9 software, leveraging its advanced search capabilities. In this study, a two-person independent screening and cross-validation mechanism was implemented, along with stringent inclusion criteria: (1) explicit labeling of the anatomical localization of joints involved in HA; (2) detailed descriptions of the pathological features, clinical manifestations, or therapeutic strategies related to joint involvement; and (3) provision of imaging or clinically verifiable data. Exclusion criteria included (1) diagnostic reports of HA that did not specify the involved joints, (2) case reports lacking anatomical localization information, and (3) review articles without primary data. Through systematic screening, 179 publications meeting the criteria were ultimately included to establish a comprehensive database of HA studies covering various joint types. This screening process ensured the completeness and accuracy of the study data in terms of anatomical localization, pathological mechanisms, and clinical interventions.
